# In Vitro Cytotoxic Effects of Celecoxib, Mefenamic Acid, Aspirin and Indometacin on Several Cells Lines

**Published:** 2016-09

**Authors:** Maryam Alsadat Hashemipour, Hoda Mehrabizadeh Honarmand, Farideh Falsafi, Mehrnaz Tahmasebi Arashlo, Saied Rajabalian, Sayed Amir Hossein Gandjalikhan Nassab

**Affiliations:** 1 Neuroscience Research Center, Kerman University of Medical Sciences, Kerman, Iran and Oral Diseases Research Center, Kerman University of Medical Sciences, Kerman, Iran.; 2 Resident of Oral and Maxillofacial Surgery and Oral Diseases Research Center, Kerman University of Medical Sciences, Kerman, Iran.; 3 Dentist, Dental and Oral Diseases Research Center, Kerman University of Medical Sciences, Kerman, Iran.; 4 Neuroscience Research Center, Kerman University of Medical Sciences, Kerman, Iran.; 5 Medical Student, Kerman Dental and Oral Diseases Research Center, Kerman University of Medical Sciences, Kerman, Iran.

**Keywords:** In Vitro, Cytotoxicity, Drug, Celecoxib, Mefenamic Acid, Aspirin, Indometacin, Cell lines

## Abstract

**Statement of the Problem:**

Use of cyclooxygenase inhibitors as chemotherapy agents has attracted the attention of a large number of investigators in recent years. Given the importance of cancer therapy, only a limited number of studies have been carried out to investigate the effects of cyclooxygenase inhibitors on specific cell lines.

**Purpose:**

This research aimed to determine the *in vitro *cytotoxic effects of cyclooxygenase inhibitors (COX-1 and COX-2 inhibitors) on KB, Saos-2, 1321N, U-87MG, SFBF-PI 39 cell lines.

**Materials and Method:**

Powders of celecoxib, mefenamic acid, aspirin and indometacin were dissolved in the appropriate solvent. The viability of cell lines was carried out by MTT (3-(4,5-Dimethylthiazol-2-yl)-2,5-Diphenyltetrazolium Bromide) assay technique. Data gathered from four separate experiments were expressed as mean±SD. Statistical significance was defined at *p*< 0.05 by using analysis of variance. Significant treatment mean values were subjected to post-hoc Tukey’s test.

**Results:**

Celecoxib showed marked cytotoxic effects on KB, Saos-2, and 1321N cells, which was significant in comparison with the control group. Celecoxib was not effective in killing U-87MG cell line. Mefenamic acid exerted cytotoxic effects on KB, Saos-2, and 1321N cells, where the viability was approximately 75%. U-87MG cells were resistant to mefenamic acid. Indometacin had the highest rate of activity on U-87MG cells, which was significant in comparison with the control group. Aspirin did not exhibit any activity on these cell lines and was not effective in killing U-87MG, KB, Saos-2, and 1321N cells.

**Conclusion:**

This research showed that celecoxib, indometacin, and mefenamic acid have the cytotoxic effects on KB, Saos-2, 1321N and U-87MG cell lines. Therefore, it appears that these drugs can be considered as anti-neoplastic agents in the experimental phase.

## Introduction

In the United States, cancer is the second leading cause of death after cardiovascular diseases. In many developing countries, the incidence of cancer is much lower most likely because of the higher death rates due to infectious diseases or traumatic injuries. The studies shows that incidence of cancer is expected due to the increases in life expectancy, increasing proportion of elderly people, and successful control of childhood diseases.[1]

At present, cancer and its treatment are considered as global problems. Complete removal of cancer without damage to the rest of the body is the goal of treatment, which sometimes can be accomplished by surgery. However, the propensity of cancers to invade the adjacent tissue or to spread to distant sites by microscopic metastasis often limits its effectiveness. Surgery often entails the removal of a wide surgical margin or a free margin. Radiation can also cause damage to normal tissues.[2] Chemotherapy is one of the most common treatment modalities for cancer. Based on the results of various researches, many chemotherapy agents have some side effects. Therefore, discovering new drugs, new properties of the existing drugs with lower side effects, and better treatment results is of great importance.[3] Some of the most promising pharmaceutical agents described to date for the prevention of cancer are the nonsteroidal anti-inflammatory drugs (NSAIDs).[4] These drugs are primarily used to treat pain and inflammation associated with arthritis.[5] NSAIDs inhibit the cyclooxygenase (COX) activity, and are also referred to as “COX inhibitors”. There exist two isoforms of COX, with distinct tissue distributions and physiological functions. COX-1 is expressed in many tissues and plays a role in production of prostaglandins that control the normal physiological processes. On the other hand, COX-2 is pro-inflammatory in nature and is expressed only in response to certain stimuli such as mitogens, cytokines, and growth factors.[6]

*In vitro*, *in vivo*, and observational evidence has demonstrated over-expression of celecoxib in solid malignancies including colon, prostate, breast, pancreas, lung, bladder, endometrium, skin basal membrane, and squamous cell malignancies. A significant relation has been established between over-expression of COX-2 and survival of patients with various cancers in retrospective studies.[4-7]

Woo *et al*. reported that mefenamic acid had an inhibitory effect on proliferation of human liver cancer cells, and induced apoptosis in them.[7] Some epidemiological studies showed that intake of aspirin decreased the risk of developing esophageal carcinoma up to 90%.[5-6,8] Some other studies revealed that indometacin impaired the development and growth of head and neck squamous cell carcinoma (HNSCC) in experimental tumor models.[9]

Given the importance of cancer therapy and the fact that only a limited number of studies have been carried out about the effects of cyclooxygenase inhibitors on these cell lines, the aim of this research was to determine the cytotoxic effects of cyclooxygenase inhibitors (COX-1 and COX-2 inhibitors) on KB (oral squamous cell carcinoma), Saos-2 (osteogenic sarcoma), 1321N (human brain astrocytoma), SFBF-PI 39 (Fibroblast-like from sheep brain) and U-87MG (malignant gliomas) cell lines *in vitro*.

## Materials and Method

**Cell culture and cell lines**

This study used KB, Saos-2, 1321N, U-87MG cell lines, which were purchased from the National Cell Bank of Iran (NCBI). These cells were immersed in RPMI 1640 medium containing 10% heat-inactivated fetal calf serum (FCS) (Serumed; Germany), 25 µg/mL of gentamycin, 2.5 µg/mL of amphotericin B, and 0.5 mg/mL of collagenase (all from Gibco-BRL; UK). After incubation for 18 h at 37°C in an environment with 5% CO_2_, the fragments were disaggregated by gentle pipetting, pelleted by centrifugation at 1500 rpm, resuspended in fresh complete growth medium, and then transferred to 25-cm[2] cell-culture flasks (Nunc; Denmark). After expansion of the culture to approximately 75-80% confluence, the cells were trypsinized and transferred to larger flasks for more proliferation. A number of vials were cryo-preserved at the third or fourth cell passages and then used in this study after defreezing.[10]

**Cytotoxicity assay**

Powder forms of mefenamic acid, indometacin, aspirin, and celecoxib were dissolved in 0.1% dimethyl sulfoxide (DMSO) to achieve a concentration of 100 μm of stock solution, which was sterilized by filtration through a 0.22-µm filter (Millipore; USA) and then stored at 4°C. Different concentrations (100, 50, 25, 10, and 5 μM) of the drugs were prepared by serial dilutions with the FCS-free medium or the medium containing 10% FCS in sterile plastic centrifuge tubes (Nunc; Denmark).

These concentrations were selected on the basis of the results of a pilot study performed in our laboratory. For each experiment, drug solutions were freshly prepared from the stock solution. In order to measure the cytotoxicity, the MTT colorimetric assay was used based on the description given by Mosmann.[11] Cultures in the exponential growth phase were trypsinized and diluted in culture medium to achieve a suspension of 1×10[6] cells/mL. One hundred μL of cell suspension was added to each centrifuge tube containing 2 mL of drug solutions. One tube containing only cells suspended in complete medium was used as control for cell viability. The tubes were then incubated for one hour at 37°C in a humidified atmosphere containing 5% CO_2_. Following the drug exposure, the cells were washed twice with 10 mL of culture medium to remove any residual drug and then resuspended in 2 mL of fresh complete growth medium. Then, 100 µL of the suspension was added to the appropriate wells of a sterile 96-well flat-bottomed microtiter plate (Nunc; Denmark). Each drug dilution was assessed in triplicate. Three wells containing only complete medium were used as blank controls for nonspecific dye reduction. The plates were then incubated at 37°C in a humidified atmosphere with 5% CO_2_ for 48-72 h. Cell viability was determined by using MTT [3-(4,5-Dimethylthiazol-2-yl)-2,5-diphenyltetrazolium bromide] assay. After 3 days of incubation, 20 μL of MTT (5 mg/mL) was added to each well. The plate was again incubated for another 4 h to allow reduction of MTT. Subsequently, the medium containing MTT was carefully aspirated from the wells. Approximately 100 μL of DMSO was added to the wells to dissolve the formazan crystals. The plate was then incubated for another 15 min. The cell viability was determined by the optical density reading of formazan solution using the ELX 800 ELISA machine.[11]

**Statistical analysis**

Data were expressed as means ± SD in four separate experiments. Statistical significance was defined at *p*< 0.05 using analysis of variance. For data analysis, post-hoc Tukey test was performed by using SPSS software, version 21.

## Results

In this research, cytotoxic effects of four non-steroidal anti-inflammatory drugs were examined on KB, Saos-2, 1321N, SFBF-PI 39, and U-87MG cell lines. Table 1 shows the effects of celecoxib on the viability of cancer cell lines at different drug concentrations. Celecoxib showed marked cytotoxic effects on KB, Saos-2, and 1321N cells, which was significant in comparison with the control group (*p*= 0.01, *p*= 0.02, *p*=0.01, respectively). Moreover, this study showed that celecoxib at concentrations ≥25 affected the KB, Saos-2, and 1321N cells (viability of cells=74.23±9.55, 87.52±8.41, 70.23± 8.06, respectively). Celecoxib was not effective in killing U-87MG cell line (*p*= 0.142).

**Table 1:** Viability of cancer cell lines treated with various concentrations of celecoxib and mefenamic acid

**Celecoxib(μm)**	**Percentage of cell viability**				
	**KB**	**Saos-2**	**1321N**	**U-87MG**	**SFBF-PI 39**
0 (control group)	98.32±8.5	103.25±7.85	92.54±9.12	103.04±5.2	107.04±1.01
6.25	95.02±5.12	105.22±1.01	98.21±2.72	101.28±3.02	101.06±1.12
12.5	81.89±6.89	98.02±8.21	90.09±8.09	100.01±2.3	104.02±2.01
25	●74.23±9.55	●87.52±8.41	●70.23±8.06	98.04±4.03	103.05±2.04
50	●65.89±5.15	●70.02±7.12	●60.09±2.54	97.54±0.92	101.04±1.01
100	●53.54±4.81	●54.65±2.13	●42.85±6.24	95.41±2.45	101.03±2.11
Mefenamic acid (μM)					
0 (control group)	102±4.15	99.02±5.25	105.01±3.12	101.58±2.26	104.03±1.01
5	98.23±3.02	90.25±2.1	107.13±0.25	105.25±4.02	101.02±1.02
10	90.54±5.1	80.12±3.25	95.21±1.42	102.32±7.12	102.06±2.11
25	●75.12±5.12	●65.02±2.12	●80.11±2.22	103.25±0.21	101.02±1.12
50	●55.02±1.35	●51.08±1.23	●60.25±4.02	101.14±4.02	105.02±1.11
100	●42.01±2.06	●40.12±1.02	●48.01±0.12	97.21±1.2	104.02±1.12
●: Means differ significantly from the control group (*p*< 0.05).					

Table 1 shows the toxic effects of mefenamic acid on the four cancerous cell lines. Mefenamic acid exerted cytotoxic effects on KB, Saos-2, and 1321N cells, where the viability was approximately 75% (concentration≥ 25). U-87MG cells were resistant to mefenamic acid, where the viability at 100 mM of drug concentration was 97.21±1.2.

Indometacin had the highest rate of activity on U-87MG cells, which was significant in comparison with the control group (*p*= 0.001). This study also showed that indometacin at concentrations≥ 25 affected U-87- MG. (Table 2)

Table 2 shows the effect of aspirin on the four cancerous cell lines. Aspirin did not exhibit any activity on these cell lines and was not effective in killing U- 87MG, KB, Saos-2, and 1321N cells (cell viability in concentrations of 100= 98.2±1.75, 97.05±2.04 and 99.26±1.75, respectively).

**Table 2:** Viability of cancer cell lines treated with various concentrations of indometacin and aspirin

**Indometacin (μM)**	**Percentage of cell viability**				
	**KB**	**Saos-2**	**1321N**	**U-87MG**	**SFBF-PI 39**
0 (control group)	98.78±5.45	99.58±4.28	108.02±1.03	109.06±2.01	106.05±1.02
5	98.54±3.02	102.45±1.08	100.05±5.29	105.03±3.12	109.07±3.11
10	101.65±3.1	100.02±2.05	105.25±6.45	98.11±4.02	108.06±2.02
25	95.17±3.32	99.55±1.54	100.48±3.42	●75.15±0.22	107.12±2.04
50	94.05±2.23	95.8±5.45	98.79±5.65	●69.05±0.18	105.04±1.01
100	95.26±2.06	94.62±6.01	99.36±4.57	●50.31±0.04	106.06±1.01
Aspirin (μM)					
0 (control group)	102.89±3.1	99.45±8.17	109.75±6.85	98.45±7.98	105.05±2.04
5	103.04±2.08	98.02±4.09	107.55±4.06	100.25±2.01	107.04±2.02
10	100.08±4.5	101.01±0.05	105.89±1.99	99.58±0.99	109.03±2.12
25	100.01±0.54	99.09±3.35	102.89±5.25	98.65±0.25	108.03±1.04
50	97.89±4.58	97.06±4.56	100.96±2.65	95.25±2.02	105.02±2.14
100	98.2±1.75	97.05±2.04	99.26±1.75	95.02±0.15	103.05±2.12
●: Means differ significantly from the control group (*p*< 0.05).					

## Discussion

More than a decade of epidemiological research suggests that people who regularly take NSAIDs have lower rates of certain precancerous conditions, cancers, and cancer-related deaths. Regular use of NSAIDs significantly reduces the risk, number, size or spread of some cancers.[5] Data are most consistent for colorectal cancer, but this decrease in risk is also seen for other cancers such as gastric cancer, esophageal cancer, breast cancer, prostate cancer, bladder cancer, and head and neck squamous cell carcinomas.[8]

Several studies have revealed that NSAIDs can even reverse the progress of some cancers. These authors believe that inhibitors of COX-1 and COX-2 can bring about a 50-93% reduction in the incidence of colorectal, prostate, lung, liver, and breast cancers.[12]

Studies have also shown that these inhibitors can increase life span of people with breast, digestive system and lung cancers.[13-14] Research shows that COX-1 and COX-2 levels are higher in tumoral cells in comparison with healthy cells.[4-6]

Atula *et al.* and Peng *et al.* found various amounts of COX-2 in hypopharynx, oropharynx and oral cancer samples. They reported that COX-2 levels increased in tumoral cells. It was concluded that the appearance of COX-2 supports the hypothesis that this enzyme might be one of the main mediators for inflammation and cancer.[15-16] Fosslein *et al.*[17] reported increased COX-1 and COX-2 levels in histological samples of patients with oral, colon and prostate cancers. Increased cyclooxygenase levels have also been reported in colon, prostate, breast, pancreases, lung, bladder, ovary, lung, stomach, brain and skin cancers. Various genetic studies have shown that there is a relationship between cyclooxygenase and prevalence of tumors.

NSAIDs were used in late 1920s as a painkiller in patients with cancer. The effects of these drugs (as chemotherapy drugs) on cancer dates back to studies carried out in 1970s. The studies showed that these inhibitors had cytotoxic and inhibitory effects on cancerous cells. In addition, large epidemiological studies have shown that these drugs might be of benefit against the development and growth of malignancies.[18]

This research showed that celecoxib and mefenamic acid had significant cytotoxicity on KB, Saos-2 and 1321N cell lines, even in low concentrations. Meanwhile, U-87MG cells were resistant to celecoxib and mefenamic acid. Indometacin had the highest rate of activity on U-87MG cells. 

Wang *et al.*[19] showed that non-cytotoxic level of Indometacin reduced the cell invasion of malignant gliomas mediated by matrix metalloproteinases (MMP-2 and MMP-9). It also lowered down the activity of MMP-2 and MMP-9, and decreased the MMP-2 secretion of cell lines; while, it is reported that celecoxib could not induce significant autophagy in U87MG cell.[20]

Liu *et al.*[21] were the first to describe tumor induction capacity of COX-2 over-expression. Celecoxib (COX-2 inhibitor) was used against colon carcinogenesis for the first time by Kawamori *et al.*[22] Matthias *et al.*[23] showed that defragmentation of DNA chains in cancerous and pre-cancerous cells decreased significantly when celecoxib was used. They concluded that this inhibitor was better than most other drugs because of its fewer side effects.

The Enhanced expression of COX-2 and up-regulation of COX-2 mRNA have been shown in colorectal, gastric, esophageal, hepatocellular, pancreatic, lung, breast, bladder, ovarian, cervical, endometrial, skin, HNSCC, and prostate cancer cells. These results suggest that enhanced expression of COX-2 might play a role in the pathogenesis of cancer; moreover, COX-2 selective inhibitors might be used for chemoprevention of cancer.^4,6 8,24^

Direct evidence of involvement of COX-2 in rat tongue carcinogenesis has been shown. Nishimura *et al.* demonstrated the suppressive effect of a selective COX-2 inhibitor, JTE522, on the growth of a xenograft of human oral SCC cell line (KB cells) implanted in the oral cavity of nude mice.[25] Another study showed over-expression of COX-2 protein in precancerous lesions and SCCs (up to 6 folds) in rat tongue induced with 4-NQQ (4-nitroquinoline-1-oxide) in a carcinogenesis rat model. These studies show that selective COX-2 inhibitors have a potential role in chemoprevention of HNSCC.[25]

Clinical investigations by Kliachkin *et al.*[26] showed that mefenamic acid decreased the activity of cathepsin D-like protease in cancerous colon tissue. The acid failed to affect the proteolytic activity of normal mucosa.

The results of this study showed that aspirin has no significant cytotoxic effects on cell lines, even in high concentrations. 

Studies carried out in British and American cancer research centers have shown that aspirin could reduce the incidence of stomach, liver, lung, colorectal, lung, skin, breast and prostate cancers.[6] Moysich *et al.*[27] studied 868 patients with lung cancer and 935 healthy individuals. They found that this drug had an inhibitory effect on lung cancer; and that those who would take this drug systematically would be less likely to develop this deadly cancer.

In addition, some epidemiological studies have shown that intake of aspirin decreased the risk of developing esophageal carcinoma up to 90%.[5-6,8, 28] Kune *et al.*[29] reported that individuals who took aspirin regularly had 40% lower risk of colon cancer compared to those who did not. Some other epidemiological studies indicated that the relative risk of developing colon carcinoma was significantly lower (about 40–50%) in patients taking aspirin or other NSAIDs.[5]

Another study demonstrated that regular daily use of aspirin was associated with 66% reduction in prostate cancer risk.[30] However, some studies demonstrated the opposite. Atula *et al.* and Jaeckel *et al.* showed that aspirin did not have any inhibitory effect on cancerous cells and it thinned the blood of patients who undergo chemotherapy, posing some problems for them.[15, 31]

One reason why the results of the present research are inconsistent with the results of other studies in relation to the effect of aspirin may be the fact that all previous studies evaluated human populations and high concentrations of aspirin (at least 300 mg/day), while in the current research, diluted aspirin powder was used with maximum concentration of 100 μL. It should be pointed out that aspirin, in high concentrations, can thin the blood, posing problems for those who undergo chemotherapy. Moreover, its effectiveness in curing this disease is a matter of controversy.

This research showed that indometacin has significant cytotoxic effects on U-87MG cell line. A study carried out by Lundholm *et al.*[32] in Sweden Cancer Center detected that simultaneous use of indometacin and chemotherapy drugs in metastatic cases decreased the pain and lengthened the life span of patients. Studies have shown that NSAIDs, including indometacin, sulindac, piroxicam, and nimesulide decreased the incidence, multiplicity and/or size of colorectal carcinomas in animal models; besides that indometacin had anti-tumoral activity on experimental esophageal tumors.[5]

Lee *et al.*[9] studied COX-2 inhibitors (indometacin and NS398) in HNSCC and found inhibition of growth of tumor cells *in vitro*, cell cycle analysis, and quantification of apoptosis. In addition, some studies revealed that COX-inhibiting NSAIDs such as piroxicam and indometacin impaired the development and growth of HNSCC in experimental tumor models.

## Conclusion

This research showed that celecoxib and mefenamic acid have the highest cytotoxic effects on KB, Saos-2, 1321N, and U-87MG cell lines. Furthermore, indometacin has the highest cytotoxic effects on U-87MG cell line. Therefore, it appears that these drugs can be introduced as anti-cancer drugs in experimental phase. 
